# Infrared and Visible Image Fusion via Residual Interactive Transformer and Cross-Attention Fusion

**DOI:** 10.3390/s25144307

**Published:** 2025-07-10

**Authors:** Liquan Zhao, Chen Ke, Yanfei Jia, Cong Xu, Zhijun Teng

**Affiliations:** 1 Key Laboratory of Modern Power System Simulation and Control & Renewable Energy Technology, Ministry of Education (Northeast Electric Power University), Jilin 132012, China; zhaoliquan@neepu.edu.cn; 2 College of Electrical and Information Engineering, Beihua University, Jilin 132013, China; jiayanfei@beihua.edu.cn; 3 School of Electronic and Information Engineering, Heilongjiang Institute of Technology, Harbin 150050, China; xucong_0803@126.com; 4 School of Electrical Engineering, Northeast Electric Power University, Jilin 132012, China; tengzhijun@neepu.edu.cn

**Keywords:** transformer, attention mechanism, deep learning, image fusion, infrared and visible images

## Abstract

Infrared and visible image fusion combines infrared and visible images of the same scene to produce a more informative and comprehensive fused image. Existing deep learning-based fusion methods fail to establish dependencies between global and local information during feature extraction. This results in unclear scene texture details and low contrast of the infrared thermal targets in the fused image. This paper proposes an infrared and visible image fusion network to address this issue via the use of a residual interactive transformer and cross-attention fusion. The network first introduces a residual dense module to extract shallow features from the input infrared and visible images. Next, the residual interactive transformer extracts global and local features from the source images and establishes interactions between them. Two identical residual interactive transformers are used for further feature extraction. A cross-attention fusion module is also designed to fuse the infrared and visible feature maps extracted by the residual interactive transformer. Finally, an image reconstruction network generates the fused image. The proposed method is evaluated on the RoadScene, TNO, and M3FD datasets. The experimental results show that the fused images produced by the proposed method contain more visible texture details and infrared thermal information. Compared to nine other methods, the proposed approach achieves superior fusion performance.

## 1. Introduction

Due to the hardware limitations of imaging sensors, a single sensor cannot capture the complete information of a scene. Infrared thermal sensors generate images by capturing thermal radiation from objects. The resulting infrared images highlight thermal targets, distinguishing them from the background. Moreover, infrared images are not affected by lighting conditions. However, they lack background details and texture information. Visible light sensors generate images by capturing reflected light. These images contain rich background details and textures. However, visible light sensors struggle to distinguish between the background and targets under poor lighting conditions. Therefore, fusing infrared and visible images allows complementary information from both sources to be combined. The fused images retain the thermal targets from infrared images and the texture details from visible images. This technique is widely used in autonomous driving [1], pedestrian re-identification [2], and military applications [3].

Over the past decade, researchers have proposed various infrared and visible image fusion methods. These methods can be broadly classified into traditional methods and deep learning-based methods. The traditional image fusion methods mainly include multi-scale transformer-based methods [4], sparse representation-based methods [5], subspace-based methods [6], and hybrid methods [7]. These methods typically involve image transformation, activity level measurement, and fusion rule design. All of these steps require manual configuration and operation, which makes the process complex and computationally expensive. Moreover, they struggle to adapt to image fusion tasks across different scenarios. Deep learning-based fusion methods rely on multi-layer deep neural networks for learning and training. They automatically extract and fuse features from data without manual intervention. This makes the fusion process simpler and more adaptable. Although deep learning-based methods have shown promising results in many scenarios, they still face significant challenges.

Most existing deep learning-based image fusion methods rely on convolution operations to extract local features and fail to capture the global context of source images. Although transformer-based methods can extract long-range contextual information, they often overlook local features. As a result, both approaches fail to establish the dependency between global and local contexts, which leads to information loss. Consequently, the fused images fail to effectively preserve visible texture details and infrared thermal radiation information. To address this issue, this paper proposes an infrared and visible image fusion network that incorporates a residual interactive transformer and cross-attention fusion. First, we design a residual dense module to extract shallow features from the source images. Second, we introduce the residual interactive transformer, which further extracts global and local contextual information from the source images and establishes interactions between the extracted information. Finally, we design a cross-attention fusion module, which exchanges and integrates complementary features extracted from infrared and visible images to generate the fused image. The fused image retains rich scene texture details and target gradient information.

The main contributions of this paper are as follows:

- This paper designs a residual dense module consisting of a mainstream and a residual stream. The mainstream incorporates dense connections, while the residual stream prevents information loss. This design enhances the extraction of shallow features from source images, facilitating further feature refinement;
- This paper proposes a residual interactive transformer consisting of three serially connected double interactive transformers with residual connections. It extracts global and local contextual information from source images and facilitates their interaction and integration, enabling more effective feature extraction;
- This paper proposes a cross-attention fusion module, which employs a cross-attention mechanism to exchange complementary features between infrared and visible images. It also generates complementary mask maps in the spatial domain, enabling the effective fusion of infrared and visible image features.

## 2. Related Work

### 2.1. Deep Learning-Based Fusion Methods

Deep learning algorithms have intense feature extraction and representation capabilities. They also demonstrate robustness and are widely used in computer vision, speech technology, and natural language processing. Infrared and visible image fusion is a fundamental and critical task in the field of computer vision [8]. Many studies have introduced deep neural networks into image fusion tasks in recent years, achieving significant results. The existing deep learning-based image fusion methods mainly address three key issues: feature extraction, feature fusion, and feature reconstruction [9]. Image fusion methods based on the use of autoencoders (AEs) for feature extraction and reconstruction first pre-train an autoencoder on large datasets. They then use manually designed fusion rules to achieve feature fusion. Xu et al. [10] proposed the CSF method, which realized the interpretability evaluation of feature map importance through deep learning for the first time. By training a binary classifier to distinguish between feature maps of visible light and infrared images, this method overcomes the limitations of traditional fusion rules. It automatically retains features that contribute significantly to classification. Li et al. [11] proposed an end-to-end deep learning-based image fusion framework, RFN-Nest. This network uses a residual fusion network and a two-stage training strategy to address the limitations of existing methods in terms of their fusion strategy design. It also employs a new loss function to preserve detailed information and significant features.

Image fusion methods based on convolutional neural networks (CNNs) are used to design network structures and loss functions. These methods perform end-to-end feature extraction, feature fusion, and image reconstruction. This enables effective image fusion. To address the issue of fusion results failing to highlight important features of source images due to insufficient consideration of the differences between multi-modal image feature maps, Xu et al. [12] proposed a feature map decomposition-based fusion network (CUFD). This method decomposes feature maps into common and unique parts and applies different fusion rules to process each part. This method better preserves the important features of source images and significantly improves the fusion results’ contrast, texture details, and object saliency. Wang et al. [13] proposed an unsupervised cross-modal generation-registration paradigm network (UMF-CMGR). This network generates pseudo-infrared images, transforming the cross-modal image registration problem into a single-modal registration problem. This solves the problem of spatial misalignment between infrared and visible light images. Liu et al. [14] proposed a novel network for multimodal image fusion. The network combines contrastive learning with a multi-level feature fusion module. It effectively reduces the information redundancy and loss in multimodal fusion.

Generative adversarial networks (GANs) have been applied in many fields, such as remote sensing imagery [15]. GAN-based methods transform the image fusion process into an adversarial process between generators. In this process, the generator generates a specific target image. The discriminator evaluates whether the target image has fused enough infrared and visible light image information. This determines whether the generated image meets the requirements for the final fused image [16]. Ma et al. [17] proposed the GANMcC method. This method introduces multi-class constraints to transform the image fusion problem into a simultaneous estimation problem with multiple distributions. The adversarial learning between the generator and discriminator balances the feature distribution of the two images. It allows the fused image to retain the high contrast of the infrared image and the rich texture details of the visible light image. To address the limitations of the existing fusion methods regarding frequency information processing, Wang et al. [18] proposed a GAN-based method, FreqGAN. This method fully utilizes low-frequency structures and high-frequency details. Approaching the frequency domain incorporates a frequency compensation generator, a mixed-frequency aggregation module, a dual-frequency constraint discriminator, and a frequency supervision loss function. This significantly improves the quality and detail representation of the fused image.

### 2.2. Vision Transformer

Vision transformer (ViT) is a neural network model based on the transformer architecture. It is used for computer vision tasks. Traditional computer vision models, such as convolutional neural networks (CNNs), have been successful in image tasks. However, CNNs have limitations, such as their weak modelling capability for long-range dependencies. ViT addresses these issues by introducing an attention mechanism to the transformer. It achieves excellent results in some vision tasks. Due to the transformer’s excellent global modelling ability and flexibility, many researchers have applied it to the image fusion field. Wang et al. [19] built a feature-encoding backbone network entirely based on the attention mechanism, without relying on CNNs. This architecture effectively models long-range dependencies, which significantly enhances its feature representation capabilities. The fused image better retains the brightness of infrared targets and the details of visible light images. Ma et al. [20] proposed a cross-domain long-distance learning framework. They designed a cross-domain module based on the attention mechanism. It includes a domain-internal fusion unit based on self-attention and a cross-domain fusion unit based on cross-attention. This effectively addresses the limitations of the existing image fusion methods regarding global context modelling and cross-domain information integration. Tang et al. [21] proposed an end-to-end fusion model based on a dual-attention transformer called DATFuse. This effectively solved the issues of long-distance dependency modelling and global context information extraction in existing fusion methods. This method retains important local features and integrates global information, generating high-quality fused images. Wang et al. [22] proposed a fusion network called AITFuse, which is based on adaptive interactive transformer learning. The network combines the strengths of CNNs and transformer. It alternately learns local and global features. This design enhances the feature representation in fusion tasks. It also addresses limitations in cross-modal interaction, local–global relation modeling, and input size constraints. To address the limitations of existing fusion methods regarding feature interaction and global information modelling, Tang et al. [23] proposed the ITFuse method. This method uses an interactive transformer framework. It can dynamically mine complementary information from different modal images. It also builds long-range dependencies through cross-modal attention and transformer blocks. This generates high-quality fused images. Cai et al. [24] proposed an intra-modal feature mixing module and a cross-modal feature mixing module to address this issue. They use residual MLP and depthwise separable convolution to implement feature mixing within and between modalities. These modules effectively integrate complementary information, improving the performance of infrared and visible light image fusion.

Existing deep learning methods, including those based on autoencoders, convolutional neural networks (CNNs), and GANs, struggle to establish long-range dependencies. This leads to the loss of global contextual information. While transformer-based methods can capture global information, their ability to extract local information is weak. Both methods overlook the dependency between global and local information in feature extraction. Therefore, this paper proposes an infrared and visible light image fusion network based on a residual interactive transformer and cross-attention fusion. It effectively integrates the interaction between global and local features and merges complementary information from infrared and visible light image feature maps. The method proposed in this paper is described in detail below.

## 3. Proposed Method

### 3.1. The Complete Network Architecture

**Figure 1:**
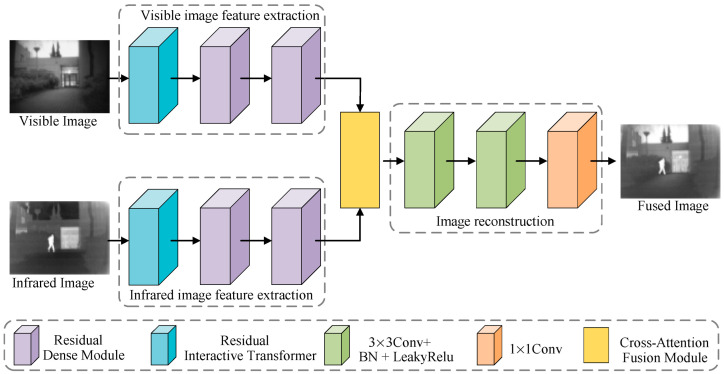
Complete architecture of infrared and visible image fusion via residual interactive transformer and cross-attention fusion.

This paper first designs the residual dense module (RDM), residual interactive transformer (RIT), cross-attention fusion module (CAFM), feature extraction network, and feature reconstruction network. These modules and networks are then used to design the infrared and visible light image fusion network. The designed network structure is shown in Figure 1. It consists of the visible light image feature extraction network, the infrared image feature extraction network, the cross-attention fusion module, and the image reconstruction network. The visible light image feature extraction network and the infrared image feature extraction network have the same structure. They both consist of one RDM module and two identical RIT modules. The feature extraction network mainly extracts features from infrared and visible light images. The RDM is mainly used to extract shallow features from the source image. The RIT module mainly extracts global and local context information from the source image. It interacts with the global and local features in the image, establishing dependencies between global and local information. The cross-attention fusion module is mainly used to fuse the features extracted by the visible light and infrared image feature extraction networks. The image reconstruction network is mainly used to reconstruct the fused image from the fused features.

### 3.2. Residual Dense Module

**Figure 2:**
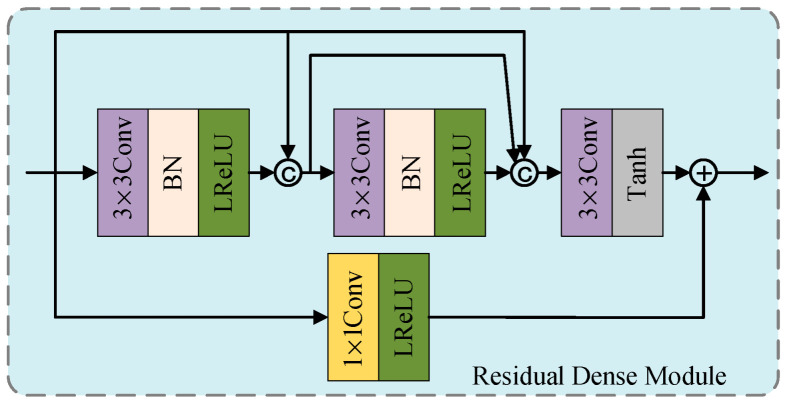
The architecture of the residual dense module.

To extract shallow features from the source image, this paper designs a residual dense module. This module, shown in Figure 2, consists of the mainstream and residual streams. The mainstream consists of two identical modules and a 3 × 3 convolutional layer with a Tanh activation function. The two identical modules contain one 3 × 3 convolutional layer, a BatchNorm layer, and a LeakyRelu activation function. We introduce dense connections in these two modules to fully utilize the features extracted by each convolutional layer. To reduce feature loss, we introduce a residual stream consisting of a 1 × 1 convolutional layer and a LeakyRelu activation function. Finally, we add and fuse the mainstream and residual stream features to obtain the final output feature map.

### 3.3. Residual Interactive Transformer

**Figure 3:**
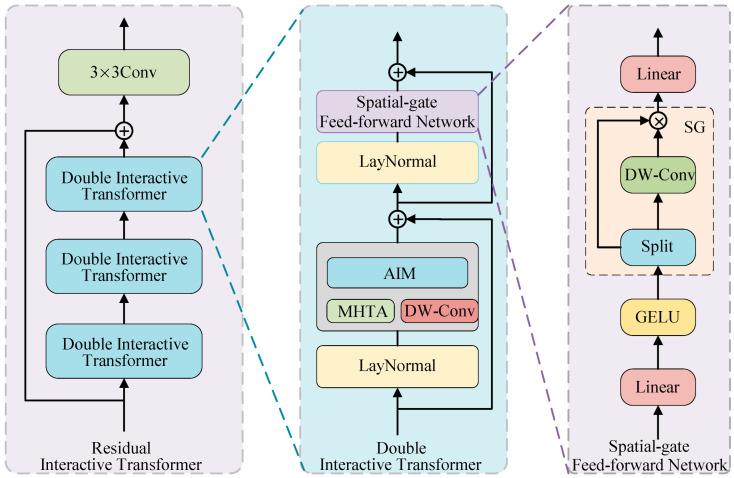
The architecture of the residual interactive transformer, double interactive transformer, and spatial gate feed-forward network.

After extracting the shallow features of the source image, we aim to effectively extract global and local context information. Additionally, we establish interactions between the global and local context information. For this purpose, we designed the residual interactive transformer module. As shown in Figure 3, the residual interactive transformer module consists of three identical double interactive transformer (DIT) modules and a 3 × 3 convolution layer. The double interactive transformer module consists of a spatial-gate feed-forward network (SGFN), two LayerNorm modules, and a mixed module comprising an adaptive interaction module (AIM), multi-head transposed attention (MHTA), and depthwise convolution (DW-Conv). Conventional feed-forward networks [25] use nonlinear activation functions and two linear projection layers for feature extraction and representation. However, they overlook the spatial and channel information in the previous layers’ self-attention layers. Therefore, we designed the spatial-gate feed-forward network (SGFN) by introducing the full spatial-gate (SG) into the feed-forward network. The SG module is a simple gating mechanism composed of depthwise convolution and element-wise multiplication. Given the input $X\in R^{H\times W\times C}$, the SGFN computation process is as follows:

(1) $$\left[X_{1}^{s},X_{2}^{s}]=split(\sigma (W_{p}^{1}X)\right)$$

(2) $$SGFN(X)=W_{p}^{2}(X_{1}^{s}⊗(W_{d}X_{2}^{s}))$$

where $W_{p}^{1}$ and $W_{p}^{2}$ represent linear projections, σ() represents the GELU function, and split() represents the splitting operation. It slices a string using a specified delimiter and returns a list. ⊗ denotes element-wise multiplication. $X_{1}^{s}$ and $X_{2}^{s}$ represent the features obtained after splitting. Our spatial gate feed-forward network can more flexibly handle spatial and channel information in the self-attention layer than conventional feed-forward networks. This enables it to capture more complex features.

**Figure 4:**
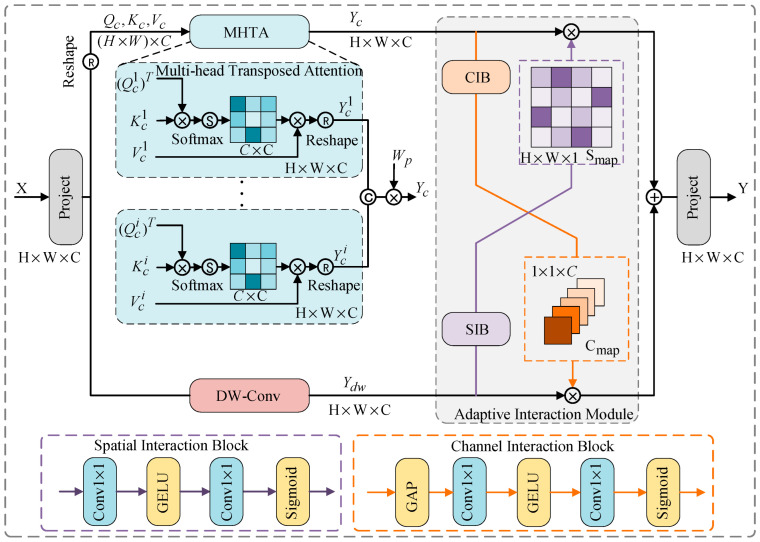
The detailed architecture of the module consisting of multi-head transposed attention, depthwise convolution, and an adaptive interaction module.

As shown in Figure 4, the multi-head transposed attention in the hybrid module is mainly used to capture the global contextual features of the image. Given an input $X\in R^{H\times W\times C}$, the matrices query, key, and value are first generated through linear projection (denoted as Q, K, and V, respectively). This process can be expressed as follows:

(3) $$Q=XW_{Q},K=XW_{K},V=XW_{V}$$

where $W_{Q}$, $W_{K}$, and $W_{V}$ are linear projections with bias omitted. Then, we perform a reshape operation on Q, K, and V. The reshaped matrices are denoted as $Q_{c}$, $K_{c}$, and $V_{c}$. Each matrix has a size of (H×W)×C. Similar to conventional multi-head attention, we divide the channels into 8 heads and then have the model learn separate attention maps, namely $Q_{c}=[Q_{c}^{1},...,Q_{c}^{i},...,Q_{c}^{8}]$, $K_{c}=[K_{c}^{1},...,K_{c}^{i},...,K_{c}^{8}]$, and $V_{c}=[V_{c}^{1},...,V_{c}^{i},...,V_{c}^{8}]$.

We transpose $Q_{c}$ and perform a dot product with $K_{c}$ to generate the transposed attention map. This attention map contains rich global information from the source image. Next, we describe the transposed self-attention generation process for the i-th head, which can be expressed as follows:

(4) $$Y_{c}^{i}=V_{c}^{i}\cdot softmax({\left(Q_{c}^{i}\right)}^{T}K_{c}^{i}/\alpha )$$

where $Y_{c}^{i}\in R^{(H\times W)\times \frac{C}{8}}$ is the output of the i-th head. α is a scaling parameter. It is not manually set. $\alpha =\sqrt{d}$, where d is the dimension of key. This metric is used to adjust the size of the dot product between ${\left(Q_{c}^{i}\right)}^{T}$ and $K_{c}^{i}$ before the Softmax function. Finally, we obtain the attention features $Y_{c}\in R^{H\times W\times C}$ by reshaping and concatenating all of the $Y_{c}^{i}$ values. This process can be expressed as follows:

(5) $$Y_{c}=MHTA(X)=concat(Y_{c}^{1},...,Y_{c}^{i},...,Y_{c}^{8})W_{p}$$

where concat() represents the concatenation of 8 attention heads. $W_{p}$ refers to the linear projection of the concatenated outputs from all attention heads into the output space $R^{H\times W\times C}$, which is the original feature space. This ensures that the input and output feature dimensions remain consistent.

The detailed structure of the adaptive interaction module is shown in Figure 4. Since the self-attention module focuses on capturing global context information of the image, we added a convolutional branch in parallel with the MHTA to extract local features of the source image. To reduce the computational complexity and improve the model’s efficiency, we used depthwise convolution instead of standard convolution to perform depthwise convolution on the value in the self-attention function, extracting local context information from the source image. We denote the output of the convolution as $Y_{dw}\in R^{H\times W\times C}$. The convolution aggregates local information from neighboring regions, while the self-attention module extracts global context information. To establish the dependency between the global feature $Y_{c}$, extracted by MHTA, and the local feature $Y_{dw}$, extracted by the convolutional branch, we use the adaptive interaction module to adaptively interact and merge the global feature $Y_{c}$ with the local feature $Y_{dw}$. The adaptive interaction module includes two types of interaction: spatial interaction and channel interaction. Both are based on the attention mechanism [26]. For the two input features, $Y_{c}$ and $Y_{dw}$, we first input the global feature $Y_{c}$ into the channel interaction block (CIB) to generate a channel attention map (denoted as $C_{map}$, size $R^{1\times 1\times C}$) along the channel dimension. The channel interaction block consists of global average pooling, two 1 × 1 convolutions, the GELU function, and the sigmoid function. This channel attention map represents the amount of information in the global feature $Y_{c}$ in the channel dimension. Similarly, we input the local feature $Y_{dw}$ into the spatial interaction block (SIB) to generate a spatial attention map (denoted as $S_{map}$, size $R^{H\times W\times 1}$) along the spatial dimension. The spatial interaction block consists of two 1 × 1 convolutions, the GELU and sigmoid functions. This spatial attention map represents the amount of information in the local feature $Y_{dw}$ in the spatial dimension. The specific representation is as follows:

(6) $$C_{map}=CIB(Y_{c}),S_{map}=SIB(Y_{dw})$$

Then, the channel attention map $C_{map}$ is element-wise multiplied by the local feature $Y_{dw}$, and the spatial attention map $S_{map}$ is element-wise multiplied by the global feature $Y_{c}$. This achieves the interaction between the global and local information. Finally, the element-wise multiplied features are fused to obtain the final feature $Y\in R^{H\times W\times C}$, and the global and local features are thus merged. The specific representation is as follows:

(7) $$Y=Y_{dw}⊗C_{map}+Y_{c}⊗S_{map}$$

where ⊗ denotes element-wise multiplication. The outputs of the two branches can be adjusted and merged through adaptive interaction to achieve better feature extraction from the source image.

Our method differs from the existing interactive transformer fusion models ITFuse and AITFuse. ITFuse uses interactive attention to combine static and dynamic information. However, it does not model dependencies between global and local features. AITFuse alternates between local and global features. It uses cascaded token-wise and channel-wise vision transformer architectures for cross-token and cross-channel interactions. Our method explicitly models global–local feature dependencies. It employs a dual interactive transformer module. We introduce multi-head transpose attention and depth-wise convolution to capture global and local features, respectively. These are dynamically fused through an adaptive interaction module.

### 3.4. Cross-Attention Fusion Moudule

**Figure 5:**
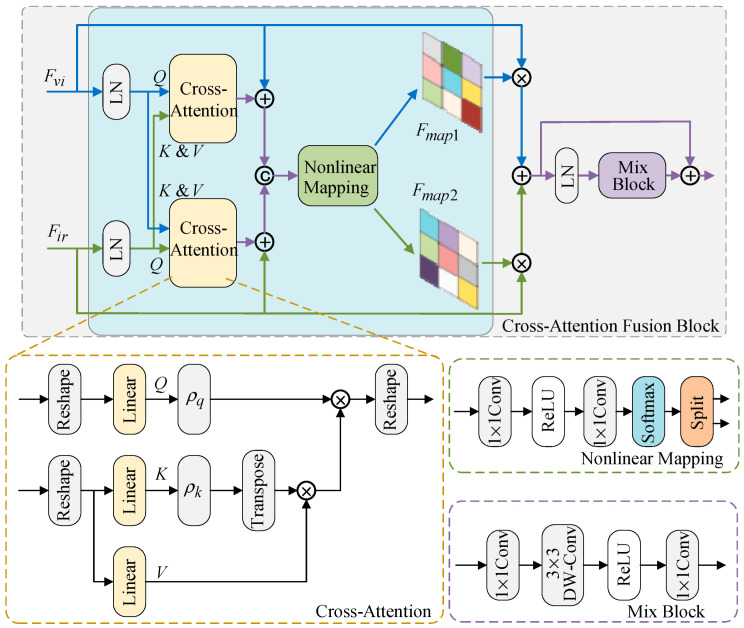
The architecture of the cross-attention fusion module.

After extracting feature maps from infrared and visible light images, we must fuse them effectively. The information in infrared and visible light images is complementary. Visible light images contain rich texture details, while infrared images provide extensive thermal radiation data. Considering the characteristics of the source images, we designed the cross-attention fusion module. The cross-attention fusion module leverages the complementarity of infrared and visible light images by exchanging their feature information, enabling effective fusion. As shown in Figure 5, the cross-attention fusion module consists of three layerNorm modules, two cross-attention units (denoted as cross-attention), a nonlinear mapping module, and a mix block.

First, we use cross-attention (denoted as CA) to exchange complementary information between the two modalities. The cross-attention process is described as follows: Given the input visible light feature map $F_{vi}\in R^{H\times W\times C}$ and infrared feature map $F_{ir}\in R^{H\times W\times C}$, we first normalize the visible light feature $F_{vi}$ and the infrared feature $F_{ir}$. Then, we reshape $F_{vi}$ into ${\hat{F}}_{vi}\in R^{(H\times W)\times C}$ and apply a linear projection to obtain query (denoted as Q). Similarly, $F_{ir}$ is reshaped into ${\hat{F}}_{ir}\in R^{(H\times W)\times C}$ and linearly projected to key and value (denoted as K and V, respectively). Similar to self-attention, we adopt a multi-head cross-attention mechanism. Therefore, Q, K, and V are divided into 8 heads, and this operator is denoted as MCA. This process can be expressed as follows:

(8) $${\hat{F}}_{vi}\;=\;reshape(LN(F_{vi})),\;{\hat{F}}_{ir}\;=\;reshape(LN(F_{ir}))$$

(9) $$Q\;=\;{\hat{F}}_{vi}W_{Q},\;K={\hat{F}}_{ir}W_{K},\;V\;=\;{\hat{F}}_{ir}W_{V}$$

(10) $$Q=[Q^{1},...,Q^{i},...,Q^{8}],K=[K^{1},...,K^{i},...,K^{8}],V=[V^{1},...,V^{i},...,V^{8}]$$

where LN() denotes LayerNorm. LayerNorm is a normalization technique used for layer-wise normalization in neural network models. $W_{Q}$, $W_{K}$, and $W_{V}$ are the linear projections of three linear layers. The cross-attention mechanism then computes the similarity between query and key. The results are weighted to obtain the attention score value, which facilitates the exchange of contextual information between the two modalities. Next, we describe the cross-attention generation process for the i-th head. It is expressed as follows:

(11) $$head^{i}=CA(Q^{i},K^{i},V^{i})=\rho_{q}(Q^{i})(\rho_{k}{\left(K^{i}\right)}^{T}V^{i})$$

(12) $$MCA(F_{vi},F_{ir})=concat(head^{1},...,head^{i},...,head^{8})$$

where $head^{i}$ represents the output of the i-th head, $\rho_{q}$ is the normalization function for feature query, and $\rho_{k}$ is the normalization function for feature key. $MCA(F_{vi},F_{ir})$ represents the final interaction feature output of the cross-attention mechanism. Similarly, we reshape the normalized infrared feature map $F_{ir}$ and project it linearly to query (denoted as Q). The normalized visible light feature map $F_{vi}$ is also reshaped and projected to key and value (denoted as K and V). These projections are then fed into the cross-attention mechanism to obtain the interaction feature output, which is denoted as $MCA(F_{ir},F_{vi})$. The cross-attention mechanism adaptively exchanges complementary features between infrared and visible light images in this process. The visible light feature map is then added as supplementary information to the interaction feature $MCA(F_{vi},F_{ir})$, which is the output of the first cross-attention module. Similarly, the infrared feature map is added to the interaction feature $MCA(F_{ir},F_{vi})$, which is the output of the second cross-attention module. The two summed features are concatenated along the channel dimension, forming a multimodal representation feature map $F_{md}\in R^{H\times W\times 2C}$. This feature map contains interaction information from both the visible light and infrared images. This process can be expressed as follows:

(13) $$F_{md}=C((MCA(F_{vi},F_{ir})⊕F_{vi}),(MCA(F_{ir},F_{vi})⊕F_{ir}))$$

where C() represents concatenation along the channel dimension, while ⊕ denotes element-wise addition. Then, we input the multimodal representation feature map $F_{md}$ into the nonlinear mapping module to generate two complementary mask maps, denoted as $F_{map1}\in R^{H\times W\times 1}$ and $F_{map2}\in R^{H\times W\times 1}$, along the spatial dimension. These masks assist in the subsequent feature fusion. They are expressed as follows:

(14) $$\left[F_{map1},F_{map2}]=NM(F_{md}\right)$$

where NM() represents the nonlinear mapping module. The nonlinear mapping module consists of two 1 × 1 convolutions, a ReLU activation function, a softmax activation function, and a split operation. We multiply the mask map $F_{map1}$ by the visible light feature map and the mask map $F_{map2}$ by the infrared feature map. The resulting feature maps are then summed to obtain the fused feature map ${\hat{F}}_{md}$. Since mask maps $F_{map1}$ and $F_{map2}$ are complementary, the complementary information from the infrared and visible light feature maps can be better integrated with their assistance. This process is expressed as follows:

(15) $${\hat{F}}_{md}=F_{map1}⊗F_{vi}⊕F_{map2}⊗F_{ir}$$

where ⊗ represents element-wise multiplication. Finally, the fused features are processed through LayerNorm and mix block to obtain the final fused feature $F_{fuse}\in R^{H\times W\times C}$. Additionally, a residual connection is introduced to prevent information loss. The process is expressed as follows:

(16) $$F_{fuse}=Mix(LN({\hat{F}}_{md}))+{\hat{F}}_{md}$$

Mix block consists of two 1 × 1 convolutions, a 3 × 3 depthwise convolution, and a ReLU activation function.

Our method differs from the existing interactive transformer fusion models ITFuse and AITFuse. We design a CAFM module that exchanges feature information between infrared and visible images via cross-attention. It also generates complementary spatial mask maps to aid fusion. Although ITFuse uses cross-modal attention, it does not include a masking mechanism. Its fusion relies more on the global reconstruction ability of transformer blocks. AITFuse achieves cross-modal feature fusion by exchanging the query, key, and value across modalities. It also does not use complementary mask maps.

### 3.5. Loss Function

In this paper, we use the intensity loss, gradient loss, and structural loss as the loss functions for the network. The specific expressions are as follows:

(17) $$L=\alpha L_{\mathrm{int}}+\lambda L_{\mathrm{grad}}+\gamma L_{\mathrm{ssim}}$$

where $L_{\mathrm{int}}$, $L_{\mathrm{grad}}$, and $L_{\mathrm{ssim}}$ represent the intensity loss, gradient loss, and structural loss, respectively. α, λ, and γ are the weights for each loss function. The intensity loss function is mainly used to measure the pixel intensity distribution between the fused image and the source image. The expression is as follows:

(18) $$L_{\mathrm{int}}=\frac{1}{HW}{||I_{f}-\max\left(I_{ir},I_{vi}\right)||}_{1}$$

where $I_{f}$ represents the fused image. H and W represent the height and width of the input image. ${\left\|\cdot \right\|}_{1}$ represents the l−1 norm. $I_{ir}$ and $I_{vi}$ represent the infrared and visible light images, respectively. max() represents the maximum pixel value of the image. The gradient loss function is mainly used to measure the gradient between the fused image and the source image. The expression is as follows:

(19) $$L_{\mathrm{grad}}=\frac{1}{HW}{\left\|\left|\nabla I_{f}\right|-\max\left(\left|\nabla I_{ir}\right|,\left|\nabla I_{vi}\right|\right)\right\|}_{1}$$

where $\left|\cdot \right|$ refers to the absolute operation and ∇ represents the gradient operator. The structural similarity loss is mainly used to measure the structural similarity between the fused image and the source image. The expression is as follows:

(20) $$L_{\mathrm{ssim}}=1-SSIM\left(I_{f},\max\left(I_{ir},I_{vis}\right)\right)$$

where SSIM() represents the structural similarity metric.

## 4. Experimental Analysis

### 4.1. Experimental Details

To evaluate the proposed method’s performance, we conducted comparative experiments on the RoadScene [27], TNO [28], and M3FD [1] datasets. The proposed algorithm was compared with CSF [10], RFN-Nest [11], GANMcC [17], CUFD [12], UMF-CMGR [13], DATfuse [21], AITFuse [22], ITFuse [23], and FreqGAN [18]. We used the mutual information (MI) [29], average gradient visual information fidelity (VIF) [30], average gradient (AG) [31], spatial frequency (SF) [32], structural similarity (SSIM) [33], and edge information Qabf [34] indices to measure the quality of the fused images. The MI index calculates the information transferred from the source to the fused image. It evaluates the fusion performance from an information-theoretic perspective. The VIF index assesses the information fidelity of the fused image from the perspective of the human visual system. The AG index measures the gradient of the fused image, describing the textures and details contained in the fused result of the source image. The SF index measures the spatial frequency information contained in the fused image. The SSIM index evaluates the fusion result’s quality by comparing the original and fused images’ structural, luminance, and contrast similarities. The Qabf index assesses the amount of edge information transferred from the source to the fused image. Higher values of these quantitative indices indicate a better image quality after fusion and an improved performance of the image fusion algorithm.

This study selected 121 pairs of infrared and visible images from the Roadscene dataset. These images include diverse scenes such as roads, vehicles, and pedestrians. The images were taken both during the day and at night. Based on this, data augmentation methods (rotation, scaling, cropping, and flipping) were used to create a training set of 9080 pairs of 64 × 64 infrared and visible image patches. During the training, we used the Adam optimizer to update the parameters. The initial learning rate was set to 0.001, with 15 epochs and a batch size of 32. The hyperparameters controlling the weights of each loss term were set to α = 20, λ = 20, and γ = 8. We used a custom-built machine assembled in China. It featured a SUPERMICRO motherboard, NVIDIA GPUs, and Intel CPUs. The system included two NVIDIA GeForce GTX 2080Ti GPUs, two Intel Xeon E5-2678 v3 CPUs, and 32 GB of RAM.

### 4.2. Comparison with State-of-the-Art Methods

#### 4.2.1. Simulation on RoadScene Dataset

**Figure 6:**
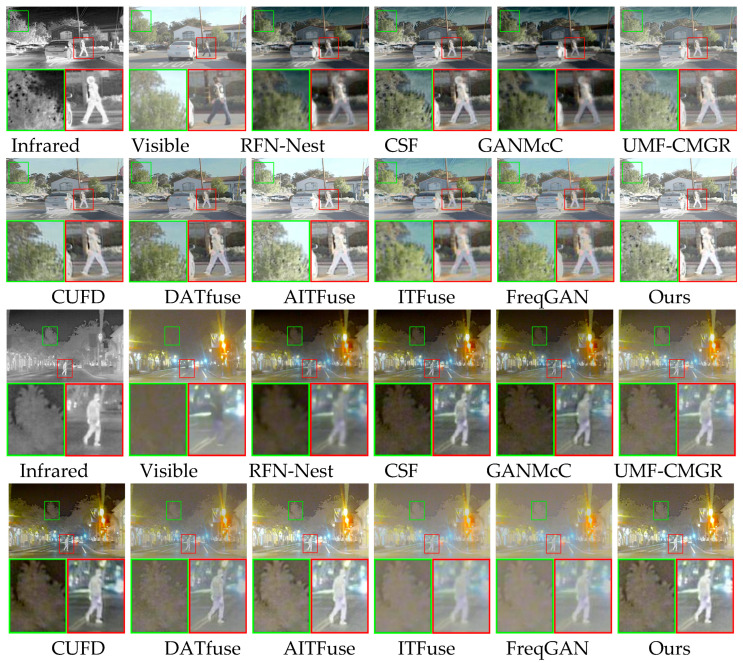
Qualitative results of different image fusion algorithms on the RoadScene dataset.

We tested the proposed method and other methods on the RoadScene dataset. We randomly selected two pairs of source images from the RoadScene dataset as test images. Figure 6 presents the two pairs of source images, the fused images, and their locally magnified versions. For better comparison, we use green and red boxes in each image to indicate areas with texture details and infrared targets, respectively. On the first set of images, the RFN-Nest method failed to integrate complementary information effectively. Its fused images have unclear infrared thermal targets and scene texture details. The fused image generated by the CSF method has precise scene texture details, but the infrared thermal target is blurred. The fused image generated by the GANMcC method shows the infrared thermal target and scene texture details as blurred. The fused images generated by the UMF-CMGR and CUFD methods have precise scene texture details. However, their infrared thermal targets contrast less than the fused images generated by the proposed method. The fused image generated by the DATfuse method is dim, and the texture details and infrared thermal targets are unclear. The fused images generated by the AITFuse, ITFuse, and FreqGAN methods and the proposed method effectively preserve visible image texture details. However, the fused image generated by the DATfuse method has a lower contrast for the infrared thermal target. In addition, the infrared thermal targets in the ITFuse and FreqGAN methods are unclear. The fused image generated by the proposed method has a higher contrast for the infrared thermal target. For the second set of images, for the RFN-Nest method, the fused image shows blurred details of both the visible light scene texture and the infrared thermal target. The fused images generated by the CSF and GANMcC methods have unclear scene texture details, and the infrared thermal targets have low contrast. The fused images generated by the UMF-CMGR and CUFD methods have high-contrast infrared thermal targets, but their scene texture details are unclear. The fused image generated by the DATfuse method has unclear visible light scene textures and infrared thermal radiation targets. The fused image generated by the AITFuse method preserves precise visible light scene texture details but has a lower contrast for the infrared thermal target. The fused images generated by the ITFuse and FreqGAN methods have blurred scene texture details and infrared thermal radiation targets. Only the fused image generated by the proposed method has precise visible light texture details and infrared thermal radiation targets.

To objectively verify the effectiveness of our method, we input all image pairs from the test set of the RoadScene dataset into the image fusion model for performance testing. The performance metrics of the fused images are shown in Table 1. As seen in Table 1, the proposed method has the highest MI value, followed by the CUFD and DATfuse methods. The proposed method has the highest SF value, followed by the CUFD and AITFuse methods. The proposed method has the highest AG value, followed by the CUFD and CSF methods. The proposed method has the highest VIF value, followed by the DATfuse and CUFD methods. The proposed method has the highest Qabf value, followed by the DATfuse and AITFuse methods. The UMF-CMGR method has the highest SSIM value, followed by the proposed and AITFuse methods. In summary, the proposed algorithm outperforms the other methods in five of the six metrics, except for SSIM, for which the result for the proposed method is lower than that of the UMF-CMGR method. This indicates that, compared to other methods, the proposed method exhibits better image fusion performance.

#### 4.2.2. Simulation on TNO Dataset

**Figure 7:**
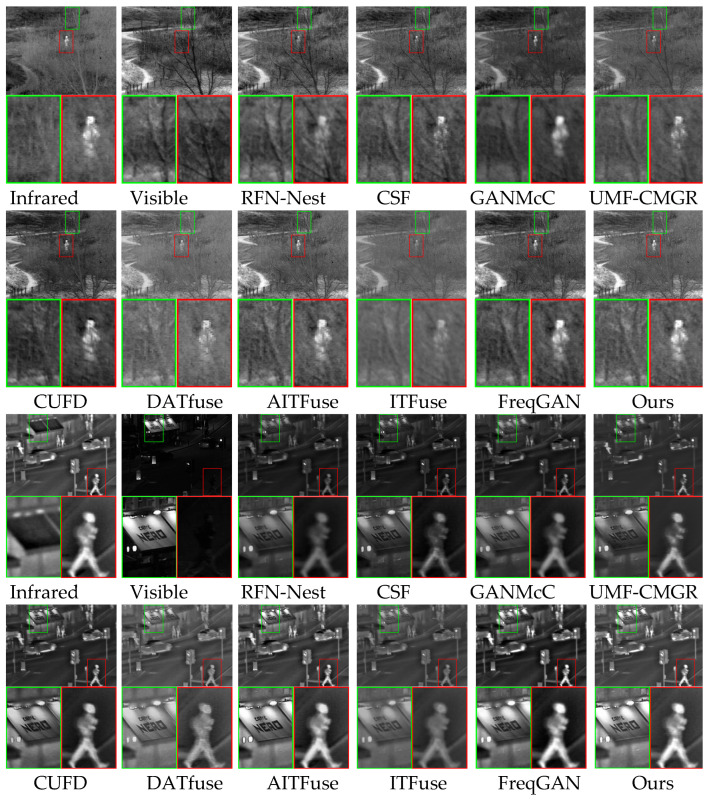
Qualitative results of different image fusion algorithms on the TNO dataset.

We also tested the proposed method and other methods on the TNO dataset. We randomly selected two pairs of source images from the TNO dataset as test images. Figure 7 presents the two pairs of source images, the fused images, and their locally magnified versions. To facilitate better comparison, we use green and red boxes in each image to denote the regions containing texture details and infrared hot targets, respectively. The first set of images shows that the fused image generated by the RFN-Nest method has unclear scene texture and infrared hot targets. The fused image generated by the CSF method displays precise scene texture details, but the infrared hot target contrast is low. The fused image generated by the GANMcC method has blurred scene texture and hot target details. The fused images generated by the UMF-CMGR, CUFD, DATfuse, and AITFuse methods have high clarity in their scene texture details, but the infrared hot target contrast is low. The ITFuse method did not effectively fuse the visible texture information and infrared radiation information, which resulted in a blurred texture and hot target details in the fused image. Only the fused images generated by the FreqGAN and proposed methods have clear infrared hot radiation targets and visible scene texture details. The second set of images shows that the fused images generated by RFN-Nest, CSF, GANMcC, and UMF-CMGR methods have unclear visible image texture details. Among them, the fused images generated by the RFN-Nest, CSF, and GANMcC methods have low clarity in the infrared hot targets. The fused image generated by UMF-CMGR has low contrast in the infrared hot targets. The fused image generated by CUFD has high contrast in the infrared hot radiation target, but its scene texture details are blurry. The fused image generated by DATfuse has unclear scene texture details and infrared hot radiation targets. The fused image generated by AITFuse has precise visible image texture details, but the infrared hot target contrast is lower compared to our method. The fused image generated by ITFuse has unclear visible scene texture details and infrared hot targets. The fused images generated by FreqGAN and the proposed method contrast highly in the infrared hot targets. However, the FreqGAN-generated image has lower clarity in its scene texture compared to our method. The fused image generated by the proposed method has high-contrast infrared hot targets and precise texture details.

To objectively evaluate the effectiveness of our method, we input all image pairs from the TNO dataset into the image fusion model for performance testing. The average performance metrics of the fused images are shown in Table 2. As shown in Table 2, the proposed method achieves the highest MI and SF values, followed by the CUFD and AITFuse methods. The proposed method has the highest AG value, followed by the CUFD and AITFuse methods. The proposed method has the highest VIF value, followed by the DATfuse and CUFD methods. The proposed method has the highest Qabf value, followed by the DATfuse and AITFuse methods. The proposed method has the highest SSIM value, followed by the UMF-CMGR and AITFuse methods. The proposed method outperforms all the other methods in the six metrics. Overall, the proposed method demonstrates superior image fusion performance compared to the other methods.

#### 4.2.3. Simulation on M3FD Dataset

**Figure 8:**
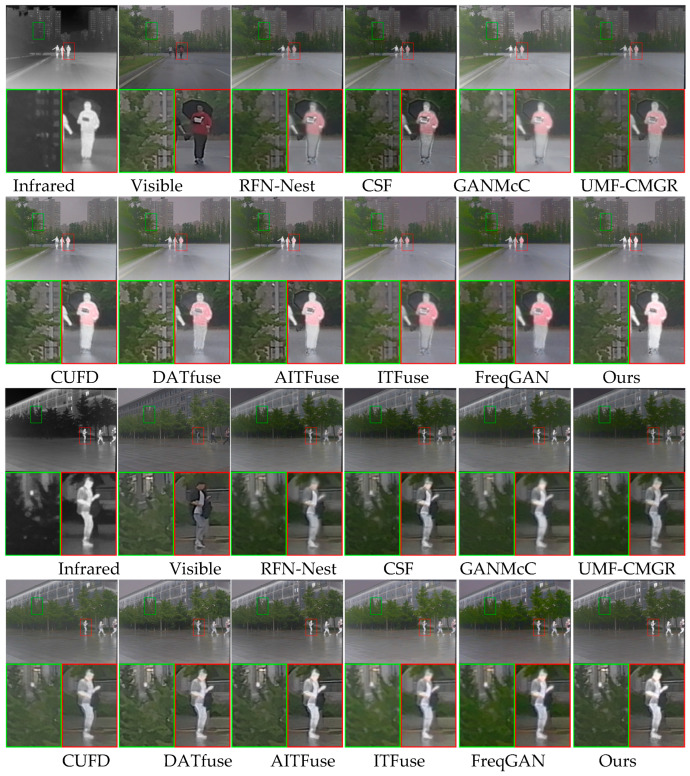
Qualitative results of different image fusion algorithms on the M3FD dataset.

We tested the proposed method and other methods on the M3FD dataset. We randomly selected two pairs of source images from the M3FD dataset as test images. Figure 8 shows the two pairs of source images, the fused images, and their zoomed-in views. For better comparison, we used green and red boxes in each image to represent areas with texture details and infrared target areas, respectively. The first set of images shows that the RFN-Nest, CSF, and GANMcC methods produced fused images that preserve visible light texture information and have relatively straightforward scene texture details. However, the fused images generated by the RFN-Nest and GANMcC methods show blurred infrared thermal targets, and the fused image from the CSF method has a low infrared target contrast. The fused image generated by the UMF-CMGR method lacks precise visible light texture details and has low infrared target contrast. The fused image generated by the CUFD method effectively preserves visible light texture information and infrared thermal radiation information. The fused image generated by the DATfuse method has blurred visible light texture and infrared thermal target details and appears relatively dark. The fused images generated by the AITFuse, ITFuse, and FreqGAN methods have precise scene texture details, but their infrared thermal target contrast is insufficient. Only our method generates fused images with precise scene texture details and high-contrast infrared thermal targets.

The second set of images shows that the fused images generated by RFN-Nest, CSF, GANMcC, and UMF-CMGR methods have relatively straightforward infrared thermal targets but lack precise scene texture details. The fused image generated by the CUFD method has clear visible light scene texture information and a clear infrared thermal target. The fused image generated by the DATfuse method has precise visible light scene texture details but a low infrared thermal target contrast. The fused images generated by AITFuse, ITFuse, and FreqGAN methods and our proposed method have precise scene texture details and high-contrast infrared thermal target details.

Similarly, to objectively validate the effectiveness of our method, we input all image pairs from the M3FD dataset into the image fusion model for performance testing. The average performance metrics of the fused images are shown in Table 3. From Table 3, it can be seen that the DATfuse method achieved the highest MI value, followed by our method and the AITFuse method. Our method achieved the highest SF and AG values, followed by the CUFD method and the AITFuse method. Our method achieved the highest VIF value, followed by the DATfuse method and the CSF method. Our method achieved the highest Qabf value, followed by the AITFuse method and the DATfuse method. Our method achieved the highest SSIM value, followed by the UMF-CMGR method and the DATfuse method. In summary, our algorithm outperforms the other methods in all six metrics except for the MI value, where its value is lower than those of the DATfuse and CUFD methods. Overall, our method demonstrates better image fusion performance compared to other methods.

## 5. Ablation Study

**Figure 9:**
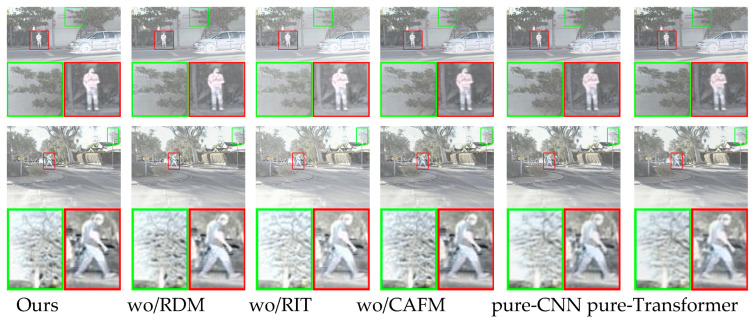
Visualization of ablation results on the RoadScene dataset. From left to right: fusion results generated by the complete method proposed in this paper, fusion results generated by the method without the RDM module, fusion results generated by the method without the RIT module, fusion results generated by the method without the CAFM module, fusion results generated by the pure-CNN backbone, fusion results generated by the pure-transformer backbone.

(1) To analyze the effectiveness of the residual dense module, residual interactive transformer, and cross-attention fusion module, we conducted the following ablation study:
  - wo/RDM: replacing the residual dense module with standard convolution for feature extraction;
  - wo/RIT: replacing the residual interactive transformer with standard convolution for feature extraction;
  - wo/CAFM: without cross-attention fusion module;
  - pure-CNN backbones: the residual dense module is used to replace all residual interactive transformers for feature extraction;
  - pure-transformer backbones: the residual interactive transformer is used to replace all residual dense modules for feature extraction;

We selected two pairs of source images from the RoadScene dataset, and the fusion results of different models are shown in Figure 9. Compared to all of the ablation models, the fusion results from our complete model exhibit precise scene texture details and infrared thermal target details, demonstrating the effectiveness of the residual dense module, residual interactive transformer, and cross-attention fusion module.

1. The ablation study of the residual dense module: The purpose of this study is to extract shallow features from infrared and visible light images. As shown in Figure 9, the fusion image generated without the residual dense module lacks the richness of scene texture information in the fusion image generated by our complete method. The results indicate that the residual dense module is crucial in generating high-quality fusion images in the network model;
2. The ablation study of the residual interactive transformer: The purpose of the residual interactive transformer is to establish an interactive relationship between the global and local context information extracted from the source images. As shown in Figure 9, the fusion image without the residual interactive transformer has blurred scene texture details and the image gradient structure is reduced. Additionally, the scene texture and infrared thermal targets cannot be distinguished. The results indicate that the residual interactive transformer is important in generating fusion images with precise scene texture details and infrared thermal targets in our network;
3. The ablation study of the cross-attention fusion module: This module aims to effectively fuse the complementary information of infrared and visible light image feature maps. As shown in Figure 9, the fusion image generated without the cross-attention fusion module has a lower contrast of the infrared thermal targets. The results indicate that the cross-attention fusion module significantly improves the quality of the fusion images generated by our method;
4. The study on pure-CNN backbones: When all residual interactive transformers are replaced with residual dense modules for feature extraction, the network becomes a pure-CNN backbone. Figure 9 shows that the fused image from the pure-CNN backbone lacks a clear visible texture. The contrast of the infrared thermal target is also reduced. These results indicate that the residual interactive transformer plays a key role in generating fused images with clear texture details and high-contrast infrared targets;
5. The study on pure-transformer backbones: When all residual dense modules are replaced with residual interactive transformers for feature extraction, the network becomes a pure-transformer backbone. As shown in Figure 9, the fused images generated by the pure-transformer backbone contain less visible texture information. The results show that the residual dense module plays an important role in generating high-quality fused images.

Next, we input all image pairs from the test set of the RoadScene dataset into different models for performance testing. The six performance metrics used for the fusion images are shown in Table 4. As seen in Table 4, our complete model generates fusion images that achieve the optimal values for four evaluation metrics compared to all ablation models. This proves the effectiveness of the residual dense module, residual interactive transformer, and cross-attention fusion module.

1. The ablation study of the residual dense module: As shown in Table 4, compared to the fusion images generated by our complete method, the method without the residual dense module achieves the highest SSIM value. However, it shows a decline in the other five evaluation metrics to varying degrees. The results demonstrate that the residual dense module effectively improves the performance of the network model;
2. The ablation study of the residual interactive transformer: As shown in Table 4, the fusion images without the residual interactive transformer show a decline in all six evaluation metrics. The decline is most significant across all metrics. The results show that the residual interactive transformer plays a key role in improving the network fusion performance;
3. The ablation study of the cross-attention fusion module: As shown in Table 4, the fusion images without the cross-attention fusion module achieve the highest Qabf value. However, there is a decline in the other five evaluation metrics. The results indicate that the cross-attention fusion module plays an important role in generating high-quality fusion images in the network model;
4. The study on pure-CNN backbones: Table 4 shows a performance drop. The fused images from the pure-CNN backbone score lower than those obtained using our full method. All six evaluation metrics show varying degrees of decline. The results indicate that the residual interactive transformer plays an important role in improving the fusion performance of the network;
5. The study on pure-transformer backbones: Table 4 shows that all six evaluation metrics drop. The fused images from the pure-transformer backbone perform worse than those from our complete method. The results show that the residual dense module can effectively improve the fusion performance.

(2) To test the effect of double interactive transformer quantity, we set the number *n* to 1, 2, 3, 4, and 5. All image pairs in the RoadScene test set were input into each model for evaluation. Table 5 shows the six evaluation metrics. When *n* = 3, the fused images do not achieve the highest SSIM, but they reach the best values in MI, SF, AG, VIF, and Qabf. Therefore, we set *n* = 3 for the double interactive transformer.

## 6. Parameter Analysis

**Figure 10:**
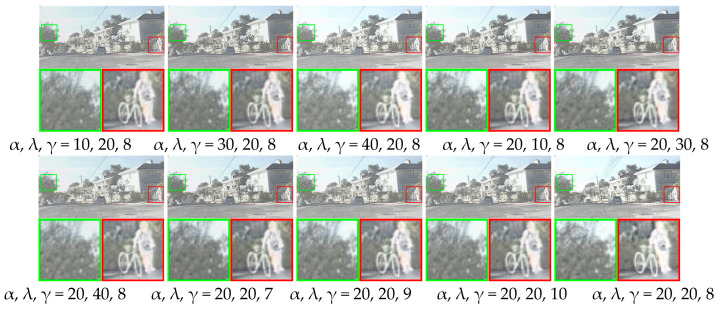
Fusion results of the method under different parameter combinations.

To analyze how hyperparameters affect the model’s performance, we conducted the following test. α is the weight for intensity loss. λ is the weight for gradient loss. γ is the weight for structural similarity loss. We tested different values for α: 10, 20, 30, and 40. During this test, λ was fixed at 20, and γ was fixed at 8. Then, we changed λ to 10, 20, 30, and 40. α was fixed at 20 and γ was fixed at 8. We also varied γ as 7, 8, 9, and 10. α and λ were fixed at 20. These settings generated different parameter combinations. One pair of source images from the RoadScene dataset was randomly selected. The fusion results under different settings are shown in Figure 10. When α was 10, 30, or 40, with λ = 20 and γ = 8, the fused images showed high-contrast infrared targets. However, the texture details were blurry. When λ was 10, 30, or 40, with α = 20 and γ = 8, the fused images had clear texture details but low contrast in their infrared targets. When γ was 7, 9, or 10, with α = 20 and λ = 20, both the texture details and infrared target contrast were unclear. Only when α = 20, λ = 20, and γ = 8 did the model produce fused images with both high-contrast infrared targets and clear visible texture details.

We tested all image pairs from the RoadScene test set on the models with different parameter combinations. The six evaluation metrics for the fused images are shown in Table 6. Table 6 shows that, when α = 20, λ = 20, and γ = 8, the model does not achieve the highest SSIM. However, it reaches the highest scores in MI, SF, AG, VIF, and Qabf. This indicates better image fusion performance. Therefore, we chose α = 20, λ = 20, and γ = 8.

## 7. Efficiency Comparison

To further validate our model, we measured its complexity using the parameter count and FLOPs. We measured the average fusion time per image on the RoadScene, TNO, and M3FD datasets to assess the model’s computational efficiency. The parameter count measures the model’s memory use. FLOPs quantify the required computations. Both reflect the complexity. Fewer parameters mean less memory usage. Lower FLOPs indicate lower computational complexity. A lower average fusion time per image means higher efficiency. We compared the RFN-Nest, CSF, GANMcC, UMF-CMGR, CUFD, DATfuse, AITFuse, ITFuse, and FreqGAN methods and our method. The results are shown in Table 7. Our method has more parameters and FLOPs than DATfuse and FreqGAN. However, it has fewer than the other six methods, ranking third. Our method ranks second in runtime on the RoadScene, TNO, and M3FD datasets. Only DATfuse is faster. Moreover, our model shows better fusion performance than DATfuse and FreqGAN.

## 8. Conclusions

This paper proposes an infrared and visible image fusion network that incorporates the residual interactive transformer and cross-attention fusion. First, we designed the residual dense module, residual interactive transformer, cross-attention module, and image reconstruction network. Then, we used the designed modules to build a complete infrared and visible image fusion network. We tested our proposed and nine other methods on the RoadScene, TNO, and M3FD datasets. Compared to the other methods, our method’s fusion images preserve the visible image’s precise texture details and the infrared image’s thermal radiation information. We also used six performance metrics—MI, SF, AG, VIF, Qabf, and SSIM—to quantitatively evaluate the performance of different methods. On the RoadScene dataset, our method had the best value for five performance metrics compared to other methods. On the TNO dataset, our method had the best value for six performance metrics. On the M3FD dataset, our method had the best value for five performance metrics compared to other methods. Overall, our method has higher performance metrics than other methods. This also means that, compared to the other methods, our method exhibits better infrared and visible image fusion performance.

## Figures and Tables

**Table 1:** Quantitative results of different image fusion algorithms on the RoadScene dataset. Red and blue indicate the best and second-best results, respectively.

Methods	MI	SF	AG	VIF	Qabf	SSIM
RFN-Nest	2.7597	8.3796	3.5902	0.4977	0.316	0.7745
CSF	2.7798	13.6697	5.4602	0.5768	0.4535	0.9543
GANMcC	2.7138	9.4399	3.9562	0.4906	0.3339	0.7958
UMF-CMGR	2.8249	11.4767	4.3617	0.5569	0.4356	0.9683
CUFD	3.7637	14.4622	5.4819	0.5831	0.4262	0.8068
DATfuse	3.7459	11.2871	3.9895	0.5955	0.4674	0.9258
AITFuse	3.7316	14.4117	5.2680	0.5633	0.4593	0.9601
ITFuse	2.594	5.0221	2.0809	0.396	0.2035	0.6488
FreqGAN	2.7569	5.7747	2.1374	0.3916	0.201	0.6239
Ours	3.7683	14.9984	5.4874	0.668	0.5315	0.9626

**Table 2:** Quantitative results of different image fusion algorithms on the TNO dataset. Red and blue indicate the best and second-best results, respectively.

Methods	MI	SF	AG	VIF	Qabf	SSIM
RFN-Nest	2.1131	5.8745	2.6693	0.5593	0.3346	0.7954
CSF	2.0630	8.7505	3.7214	0.5862	0.3963	0.9363
GANMcC	2.2679	6.1610	2.5442	0.5297	0.2806	0.8438
UMF-CMGR	2.2092	8.1747	2.9727	0.5946	0.4104	1.0107
CUFD	3.4071	9.9678	4.0435	0.6667	0.3910	0.8076
DATfuse	3.1270	9.6057	3.5602	0.683	0.4997	0.9379
AITFuse	3.3258	9.8566	3.9624	0.6532	0.4876	1.0069
ITFuse	2.1492	3.9478	1.6959	0.4462	0.2076	0.7233
FreqGAN	2.4291	4.1177	1.6108	0.4425	0.1865	0.6736
Ours	3.7109	10.5687	4.0814	0.7950	0.5438	1.0512

**Table 3:** Quantitative results of different image fusion algorithms on the M3FD dataset. Red and blue indicate the best and second-best results, respectively.

Methods	MI	SF	AG	VIF	Qabf	SSIM
RFN-Nest	2.8497	7.7418	2.8696	0.5782	0.4027	0.7849
CSF	2.8694	9.6908	3.525	0.6265	0.4644	0.9064
GANMcC	2.7648	7.4739	2.6841	0.5368	0.3158	0.8192
UMF-CMGR	3.0393	8.7829	2.9443	0.6055	0.3947	0.9194
CUFD	3.7450	10.8490	3.8212	0.5623	0.3907	0.7854
DATfuse	4.1299	10.4667	3.4430	0.6444	0.4936	0.9193
AITFuse	3.9831	10.412	3.6911	0.5948	0.5988	0.9071
ITFuse	2.7781	5.1975	1.9410	0.4504	0.1960	0.7165
FreqGAN	3.1391	4.7506	1.6387	0.4124	0.1433	0.6093
Ours	4.0136	13.5104	4.6549	0.7682	0.6003	0.9926

**Table 4:** Quantitative evaluation results of the ablation study on all images in the RoadScene test dataset. Red indicates the best result.

	MI	SF	AG	VIF	Qabf	SSIM
wo/RDM	3.4304	14.3128	5.3157	0.6309	0.5155	0.9758
wo/RIT	3.1398	13.8430	5.0639	0.5678	0.4764	0.9126
wo/CAFM	3.7347	14.6095	5.4633	0.6627	0.5467	0.9618
pure-CNN	3.3436	13.7614	5.1882	0.5571	0.4464	0.9471
Pure-transformer	3.2154	13.0047	4.9606	0.5820	0.4477	0.9749
ours	3.7683	14.9984	5.4874	0.6680	0.5315	0.9626

**Table 5:** Quantitative evaluation results on all RoadScene test images with different numbers of double interactive transformer layers. Red indicates the best result.

*n*	MI	SF	AG	VIF	Qabf	SSIM
1	3.0924	10.7666	4.1437	0.5241	0.4034	0.8998
2	3.5052	11.9063	4.5684	0.5872	0.4527	1.0069
3	3.7683	14.9984	5.4874	0.6680	0.5315	0.9626
4	3.5114	12.4069	4.6689	0.5835	0.4550	0.9632
5	3.3465	13.3152	5.113	0.5751	0.4559	0.9669

**Table 6:** Quantitative evaluation results of all RoadScene test images under different parameter combinations. Red indicates the best result.

α, λ, γ	MI	AG	VIF	Qabf	SSIM
10, 20, 8	3.2023	4.7193	0.5396	0.4484	0.9779
30, 20, 8	3.3806	4.6288	0.5548	0.4387	0.9782
40, 20, 8	3.5584	4.6197	0.5859	0.4433	0.9482
20, 10, 8	3.5366	4.7582	0.5875	0.4424	1.025
20, 30, 8	3.3382	4.9995	0.5646	0.4595	0.9749
20, 40, 8	3.1524	4.7623	0.5333	0.4456	0.9514
20, 20, 7	3.4037	4.8326	0.5694	0.4524	0.9904
20, 20, 9	3.3322	4.8708	0.5566	0.4510	0.9938
20, 20, 10	3.3480	4.8318	0.5539	0.4497	0.9968
20, 20, 8	3.7683	5.4874	0.668	0.5315	0.9626

**Table 7:** Parameter counts, FLOPs, and runtimes of different methods on the RoadScene, TNO, and M3FD datasets. Red and blue indicate the best and second-best results, respectively.

Methods	Size(M)	FLOPs(G)	RoadScene(s)	TNO(s)	M3FD(s)
RFN-Nest	1.0225	32.85	0.2746	0.2361	0.2962
CSF	1.2277	70.32	2.6985	2.2154	2.9851
GANMcC	0.7251	23.59	0.1965	0.1852	0.2295
UMF-CMGR	0.6826	16.03	0.1953	0.1719	0.2082
CUFD	0.9854	29.32	0.2484	0.2043	0.2879
DATfuse	0.0886	3.79	0.1276	0.1143	0.1489
AITFuse	0.7633	10.56	0.1682	0.1366	0.1935
ITFuse	0.2336	7.96	0.1523	0.1371	0.1625
FreqGAN	0.1026	4.35	0.1432	0.1221	0.1601
Ours	0.1282	4.46	0.1309	0.1206	0.1573

## Data Availability

Data are contained within the article.
